# From nano- to micrometer scale: the role of microwave-assisted acid and alkali pretreatments in the sugarcane biomass structure

**DOI:** 10.1186/s13068-018-1071-6

**Published:** 2018-03-22

**Authors:** Augusta Isaac, Jéssica de Paula, Carlos Martins Viana, Andréia Bicalho Henriques, Angelo Malachias, Luciano A. Montoro

**Affiliations:** 1 Department of Metallurgical and Materials Engineering, Universidade Federal de Minas Gerais, Belo Horizonte, 31270-901 Brazil; 2 Microscopy Center, Universidade Federal de Minas Gerais, Belo Horizonte, 31270-901 Brazil; 3 Mining Engineering Department, Universidade Federal de Minas Gerais, Belo Horizonte, 31270-901 Brazil; 4 Department of Physics, Universidade Federal de Minas Gerais, Belo Horizonte, 31270-901 Brazil; 5 Department of Chemistry, Universidade Federal de Minas Gerais, Belo Horizonte, 31270-901 Brazil

**Keywords:** Sugarcane bagasse, Microwave-assisted pretreatment, Atomic force microscopy, X-ray diffraction, Cellulose microfibril

## Abstract

**Background:**

To date, great strides have been made in elucidating the role of thermochemical pretreatments in the chemical and structural features of plant cell walls; however, there is no clear picture of the plant recalcitrance and its relationship to deconstruction. Previous studies precluded full answers due to the challenge of multiscale features of plant cell wall organization. Complementing the previous efforts, we undertook a systematic, multiscale, and integrated approach to track the effect of microwave-assisted H_2_SO_4_ and NaOH treatments on the hierarchical structure of plants, i.e., from a nano- to micrometer scale. We focused on the investigation of the highly recalcitrant sclerenchyma cell walls from sugarcane bagasse.

**Results:**

Through atomic force microscopy and X-ray diffraction analyses, remarkable details of the assembly of cellulose microfibrils not previously seen were revealed. Following the H_2_SO_4_ treatment, we observed that cellulose microfibrils were almost double the width of the alkali pretreated sample at the temperature of 160 °C. Such enlargement led to a greater contact between cellulose chains, with a subsequent molecule alignment, as indicated by the X-ray diffraction (XRD) results with the conspicuous expansion of the average crystallite size. The delignification process had little effect on the local nanometer-sized arrangement of cellulose molecules. However, the rigidity and parallel alignment of cellulose microfibrils were partially degraded. The XRD analysis also agrees with these findings as evidenced by large momentum transfer vectors (*q* > 20 nm^−1^), interpreted as indicators of the long-range order of cell wall components, which were similar for all the studied samples except with application of the NaOH treatment at 160 °C. These changes were followed by the eventual swelling of the fiber cell walls.

**Conclusions:**

Based on an integrated approach, we presented multidimensional architectural models of cell wall deconstruction resulting from microwave-assisted pretreatments. We provided direct evidence supporting the idea that hemicellulose is the main barrier for the swelling of cellulose microfibrils, whereas lignin adds rigidity to cell walls. Our findings shed light on the design of more efficient strategies, not only for the conversion of biomass to fuels but also for the production of nanocellulose, which has great potential for several applications such as composites, rheology modifiers, and pharmaceuticals.

## Background

Lignocellulosic biomass is considered to be a potential renewable and environmentally friendly feedstock for sustainable production of biofuels to meet global concerns of greenhouse gas emissions and depletion of fossil fuels [1, 2]. To convert biomass into liquid fuels, sugars that are stored as high molecular weight polymers in the cell walls must be deconstructed and released into solution as monosaccharides [3].

One of the key barriers to cost-effective industrial conversion of cellulosic biomass to liquid fuels is the recalcitrance of plants, which refers to the natural resistance against assault on the plant’s structural sugars from the microbial and animal kingdoms [2]. Plant cell walls are macrometer-sized in nature and their composition and organization vary significantly throughout the hierarchical structure of plants [4]. This complexity and the highly cross-linked nature of cell walls likely contributes to the plant’s recalcitrance, particularly the presence of a matrix of hemicellulose and lignin that encapsulates and restricts access to cellulose microfibrils [4, 5].

Several investigations have been driven by the search of pretreatment methods that can increase biomass digestibility for subsequent enzymatic hydrolysis [6, 7]. Many pretreatments have been proposed and investigated, such as alkaline and dilute acid [8–11], steam explosion [12], ammonia fiber expansion [13, 14], organic solvent [15], liquid hot water [16–19], and ionic liquid pretreatments [20–26]. To achieve high cellulose conversion, most thermochemical pretreatments are carried out at relatively high reaction severity, which requires high temperatures or high operating pressures. As an alternative to conventional heating, microwave radiation has provided impressive acceleration and higher yields under milder reaction conditions [27, 28]. Currently, great efforts have been taken to improve the efficiency and robustness of these approaches to meet the requirements for the successful implementation of cellulosic biorefineries. Though significant strides have been made in elucidating the effect of the pretreatments on the accessibility of cellulose to enzymes, a comprehensive picture of plant cell wall deconstruction is still unclear. Previous studies precluded full answers due to the challenge of multiscale features of plant cell wall organization [13]. Complementing the previous efforts, there is a need for a systematic, multimodal, and integrated approach to investigate biomass recalcitrance and its relationship to deconstruction over this whole scale range. This is especially true for microwave irradiation-assisted methods.

Accordingly, the objective of this study was to explain the chemical and ultrastructural organization of cell walls, and more importantly, how this architecture is modified by pretreatments, using several imaging and characterization techniques. Here, we examined the effect of microwave-assisted H_2_SO_4_ and NaOH pretreatments with different severities (at temperatures of 130 and 160 °C) on sugarcane bagasse cell walls.

## Results

A multimodal and integrated approach was applied to investigate the spatial hierarchical structure of sugarcane bagasse (SB) cell walls before and after microwave-assisted thermochemical pretreatments. The effect of sulfuric acid (mw-acid) and sodium hydroxide solutions (mw-alkali) at two different temperatures (130 and 160 °C) on cell walls was examined.

### Chemical changes in sugarcane bagasse by pretreatments

**Fig. 1:**
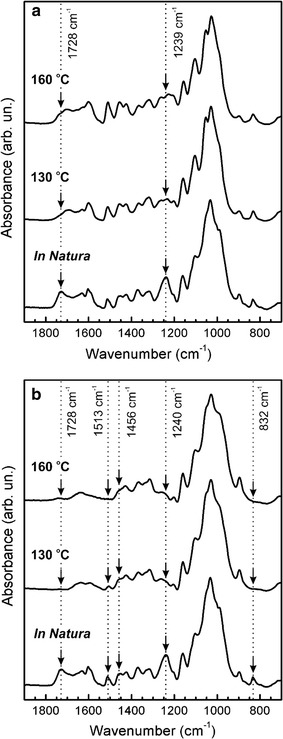
FTIR spectrum of sugarcane bagasse pretreated for 30 min under microwave processing. **a** H_2_SO_4_ pretreatments at 130 and 160 °C and **b** NaOH pretreatments at 130 and 160 °C

Cellulose is a homopolysaccharide composed of a β-d-glucopyranose unit linked together by (1→4)-glycosidic bonds [27]. The solid residues of SB were qualitatively analyzed by FTIR at two different temperatures (130 and 160 °C) after 30 min of mw-H_2_SO_4_ and mw-NaOH pretreatments (Fig. 1). Figure 1b shows sharp bands between 850 and 1200 cm^−1^ that are associated with the cellulose and hemicellulose structures [29, 30]. This spectral region was not significantly modified upon NaOH treatment, which indicates that the cellulose and hemicellulose structures were not considerably affected by this chemical processing. However, this spectral range was modified upon mw-acid pretreatment. Figure 1a shows that the peak at 1239 cm^−1^, attributed to acetyl C–O stretching of the hemicellulose structure, was almost absent after the acid treatment. This implies that the hemicellulose was effectively deacetylated [26]. The peak at 1728 cm^−1^ appears from the complex linkages between hemicellulose and lignin, such as ester-linked acetyl, feruloyl, and *p*-coumaroyl groups [31]. For the acid-pretreated SB particles, there was no absorbance at this position, indicating that these linkages were broken. Throughout the heating process, polar groups of a molecule are largely affected by microwaves, whereas less polar parts (i.e., lignin) are less interactive [28].

Lignin has characteristic absorbance bands from 1450 cm^−1^ to approximately 1730 cm^−1^ [29]. Typical bands at 1456, 1511 and 1600 cm^−1^ can be used as a lignin fingerprint on the samples [29]. The absorption at 1456 cm^−1^ is attributed to a C–H bending, and the band at 1513 cm^−1^ is related to C=C aromatic stretches [32]. Furthermore, typical lignin C–H stretching is observed at approximately 832 and 1240 cm^−1^. The lignin fingerprint was clearly observed in the FTIR spectrum of the control and acid-pretreated samples, but not for those obtained for the NaOH pretreated samples (Fig. 1b). This is clear evidence that lignin was efficiently removed. Furthermore, there was no absorbance at 1728 cm^−1^ after the alkali pretreatments, indicating that the linkages between hemicellulose and lignin were broken. In summary, both H_2_SO_4_ and NaOH could effectively degrade hemicellulose and break or weaken the linkages between lignin and hemicellulose. Furthermore, NaOH could effectively remove lignin, whereas H_2_SO_4_ removed more hemicellulose.

### Exploiting plant cell wall architecture by multiscale imaging techniques

#### Ultrastructural and chemical characteristics of cell walls

The effects of different pretreatments on the plant tissues and cell walls at the micro- and nanometer scales were investigated using scanning and transmission electron microscopy. In addition, cytochemical staining was applied to localize lignin within cell walls. A range of cytochemical stains has been reported to be useful for detecting and localizing lignin by TEM, including mercury, bromine, and potassium permanganate [33]. Here, lead citrate was used as staining agent to improve image contrast.

**Fig. 2:**
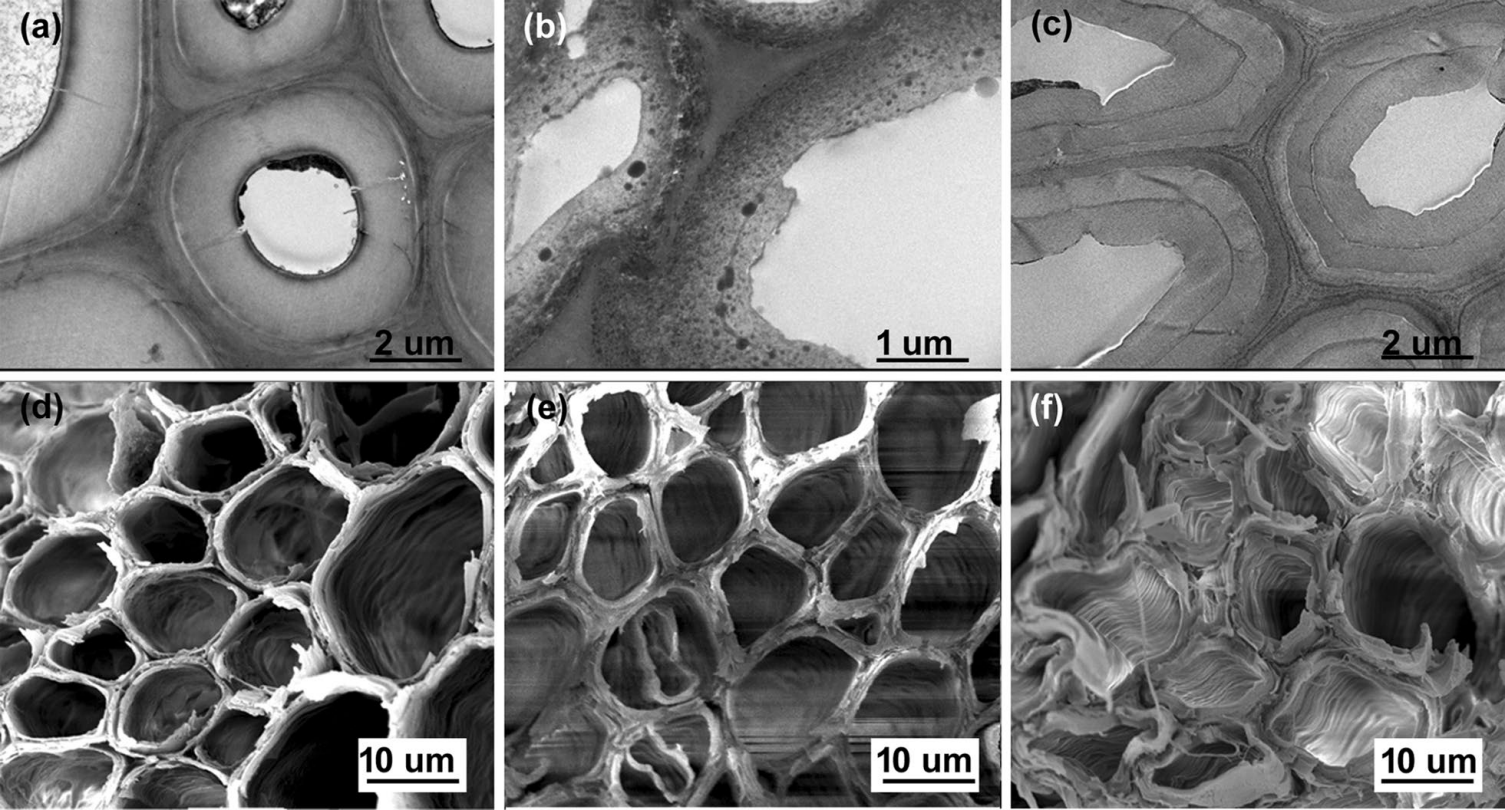
TEM and SEM images of sugarcane bagasse sclerenchyma cells. TEM images of the control sample before pretreatment (**a**) and samples pretreated at 160 °C for 30 min in 1% H_2_SO_4_ (**b**) and 1% NaOH (**c**). SEM images of the untreated substrate (**d**) and samples pretreated at 160 °C for 30 min in 1% H_2_SO_4_ (**e**) and 1% NaOH (**f**)

Figure 2 displays representative images of the TEM and SEM performed on sectioned untreated samples and microwave-assisted pretreated SB samples at the temperature of 160 °C. The structure of the mw-acid and mw-alkali substrates at this temperature on TEM images exhibited significant differences from that of the original SB (Fig. 2). The intercellular layers of the untreated SB could be clearly distinguished between the middle lamella (ML), primary cell wall (PW), and secondary cell wall (SW). The cell corner (CC) could also be easily identified (Fig. 2a). As expected, the highest lignin content was localized at the CC, followed by the ML [9]. The consecutive PW layer appeared more opaque to the electron beam as compared to the secondary wall, which provides direct evidence of the greater amount of lignin content (Fig. 2a). The transition between PW and SW was more electron-lucent than any other region of the cell wall and the outermost layer (waxy cover) was distinctly denser than the inner secondary wall (Fig. 2a).

From our FTIR results, we could confirm that the mw-acid pretreatments with different severities effectively removed hemicellulose, which might have altered the interaction between cellulose and other components of the secondary walls, especially lignin. In addition to the effect of hemicellulose removal on the interaction between cellulose and lignin, lignin undergoes extensive modification of its native state and may be forced to migrate and coalesce forming small droplets (10–100 nm) when the pretreatment temperature exceeds the lignin melting temperature (≈ 120 °C) [33, 34]. These factors likely explain the formation of lignin globules (up to tens of nanometers in diameter) in the acid-pretreated cell walls (Fig. 2b). We presume that lignin originally present in CCs and CML extruded outwards through secondary walls and deposited in specific ultrastructural layers. Previous studies have reported that the deposition of lignin droplets on the surface of the residual biomass acts as a physical barrier and causes nonproductive binding to hydrolytic enzymes [33, 35, 36]. However, some authors state that the process of lignin melting and migration may increase the accessibility of cellulose microfibrils deep within cell walls [34, 37]. The key mechanisms responsible for inhibition of cellulose hydrolysis by lignin, nonspecific binding or surface blockage or both, are believed to depend on the chemical nature and particle size of lignin polymer molecules [34, 37]. Further studies are still necessary to clarify this issue.

Figure 2c displays the cell wall organization of the mw-alkali treated SB, which differs considerably from that observed for the acid-pretreated sample. The SB morphology treated with dilute NaOH solution was more preserved in terms of its physical integrity. However, a swelling of the secondary walls was clearly observed which resulted in a conspicuous increase in wall thickness. Moreover, the alkali solution induced a considerable reduction in lignin throughout cell walls and the removal of the waxy layer (Fig. 2c). Several authors reported the swelling of cell walls in lignocellulosic materials after alkali pretreatments [38, 39]. For instance, the cell wall swelling was observed for barley straw (*Hordeum vulgare*) after dilute NaOH pretreatments (0.5 to 2% w/v) at boiling temperature with low residence time (10 min) [38]. Visualization of dynamic changes in poplar cell walls during sodium hydroxide pretreatment (2% w/v, 121 °C) confirmed that swelling occurs primarily in secondary walls and alkali had little effect on the cell corner middle lamella [39].

Figure 2d–f provide information on the morphological heterogeneity across the sectioned SB substrates. The plant tissue architecture of untreated substrates is highly ordered, consisting of sclerenchyma fibers surrounding vascular bundles (Fig. 2d). The SEM image of the mw-acid-pretreated SB shows that the delamination of the secondary walls was extensive enough to nearly detach entire layers (Fig. 2e), which increases the available surface area and subsequent hydrolytic enzyme accessibility [40]. The delamination event may derive from the depolymerization of hemicelluloses and relocation of lignin [41]. From the SEM image of the mw-alkali treated substrates (Fig. 2f), a helical orientation of cellulose microfibrils on the cell wall surface could be visualized on the inner surface of the cell walls. The helical windings of cellulose microfibrils are usually found in the middle secondary wall of lignocellulosic fibers [42]. This indicates that the mw-alkali pretreatments removed the innermost layers of the fiber cell walls.

#### Macromolecular ultrastructure of the sugarcane bagasse cell walls

**Fig. 3:**
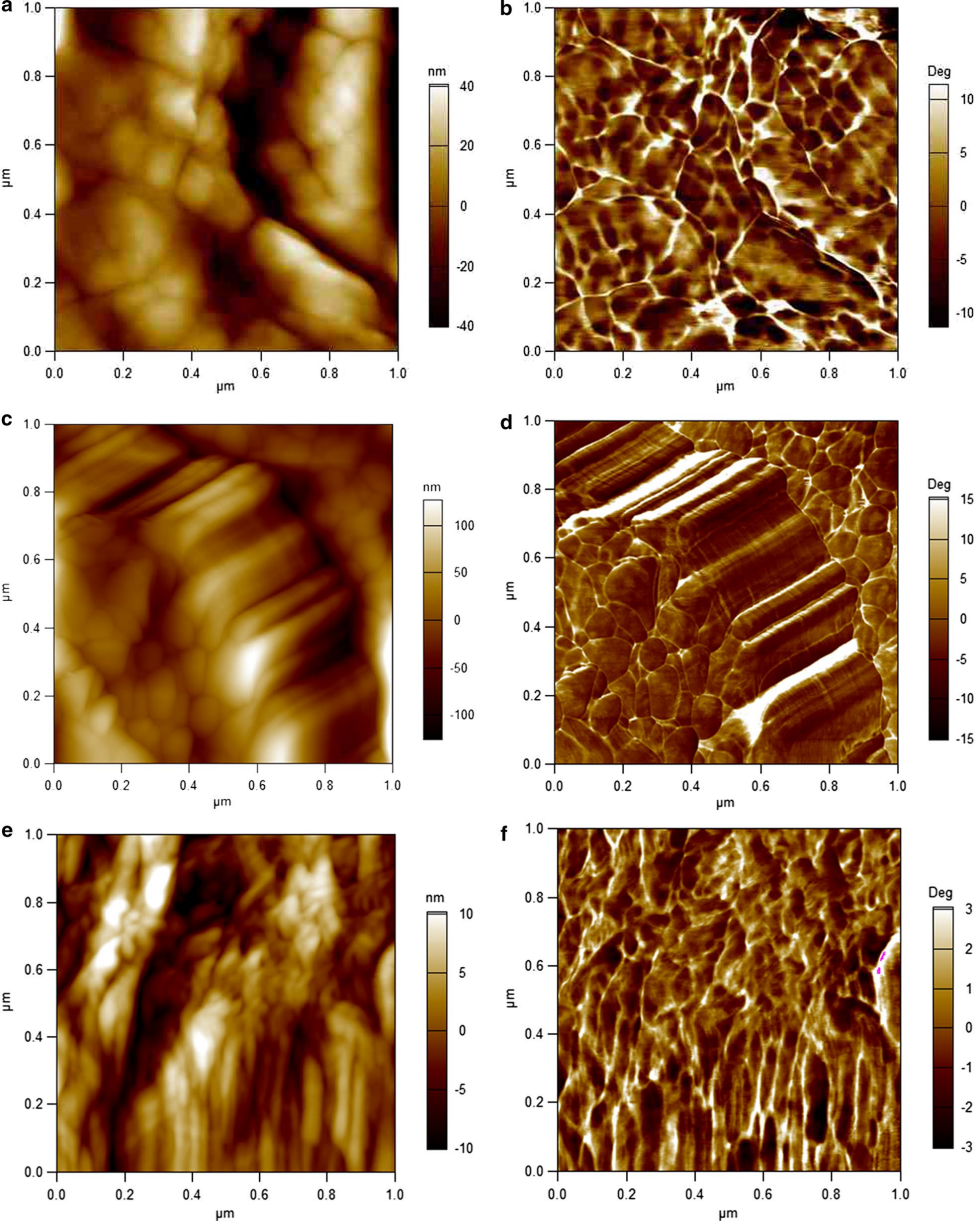
Comparison of the macromolecular ultrastructure of the untreated and pretreated sugarcane bagasse cell walls. AFM amplitude and phase images of the untreated (**a**, **b**), mw-acid (**c**, **d**), and mw-alkali pretreated SB (**e**, **f**) at the temperature of 160 °C for 30 min. Scanned area of 1 × 1 µm

To further identify the cell wall architectural features that may contribute to differences in digestibility and enzymes accessibility, we used atomic force microscopy (AFM) to image cell walls at the nanometer scale. Amplitude and phase images were captured to present modifications in sclerenchyma cells after the microwave-assisted pretreatments. Representative AFM images selected from over 200 images of untreated and pretreated SB cell walls at the temperature of 160 °C are shown in Fig. 3.

**Fig. 4:**
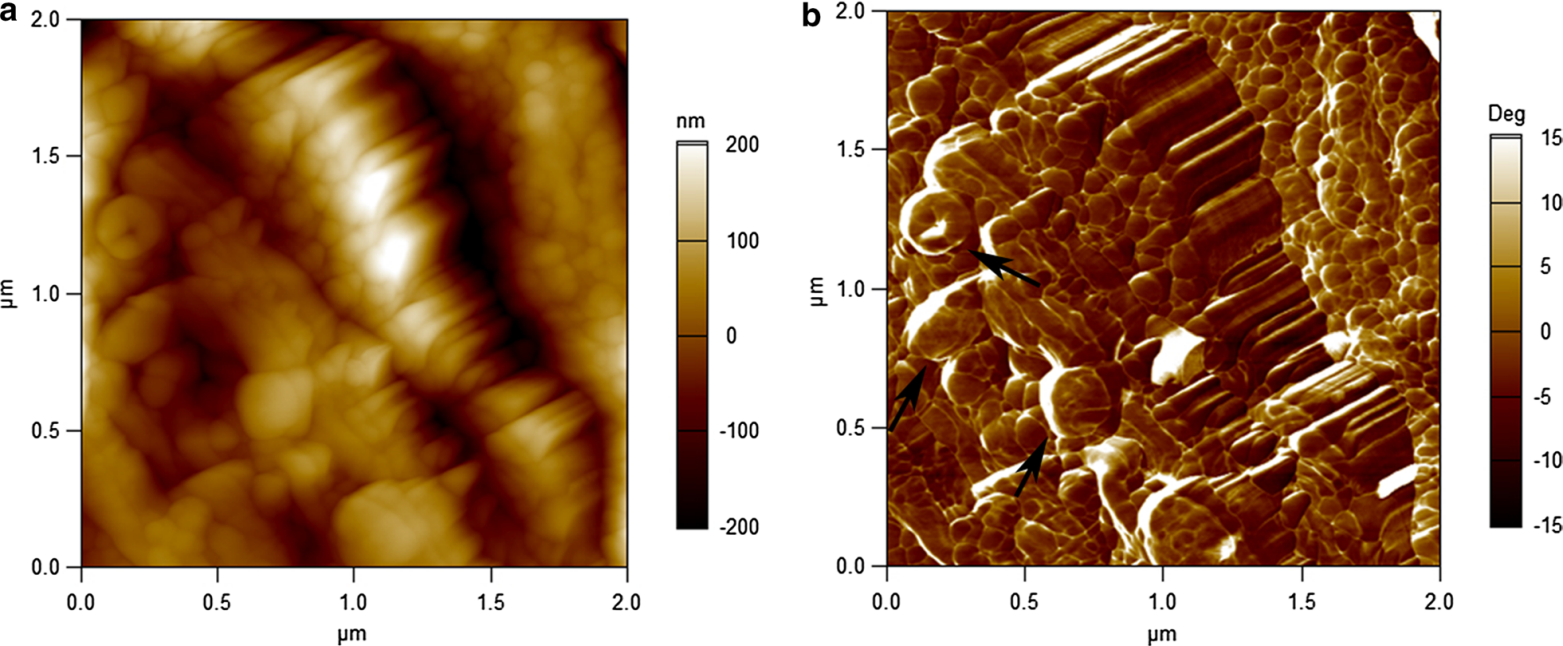
Globular structures on the cell wall surface of the mw-acid-pretreated fibers. AFM amplitude (**a**) and phase (**b**) images of the mw-acid at the temperature of 160 °C for 30 min. Scan area of 2 × 2 µm. Lignin droplets are indicated by black arrows

The surface roughness of the native sclerenchyma cells was significantly different from the pretreated cells and was more evident with increasing pretreatment severity (Fig. 3). The AFM image of the untreated SB indicates a rough cell surface that masks any fibrillar feature, indicating that the cellulose microfibril assemblies were coated with matrix polymers (Fig. 3a and b). The mw-acid-pretreated fibers, however, exhibited a highly dense network of cellulose microfibrils oriented in a specific direction. The average width of these microfibrils was approximately 100 nm (Figs. 3c and d). Moreover, globular structures with a diameter of approximately 100 nm could be visualized on the cell wall surface of the acid-pretreated fibers, which are characteristic of lignin droplets [33, 43–45] (Fig. 4). Interestingly, the average width of the cellulose microfibrils of the mw-alkali pretreated sugarcane bagasse was significantly smaller than those visualized after the acid pretreatment; furthermore, the microfibrils were less orderly packed (Fig. 4e and f). Our results agree well with recent studies of the macromolecular ultrastructure of acid-pretreated substrates in similar conditions [41, 43]. More specifically, Zubov et al. [43] reported that the width of cellulose microfibrils and the diameter of lignin droplets in wood were approximately 20–50 nm and 5–60 nm, respectively.

**Fig. 5:**
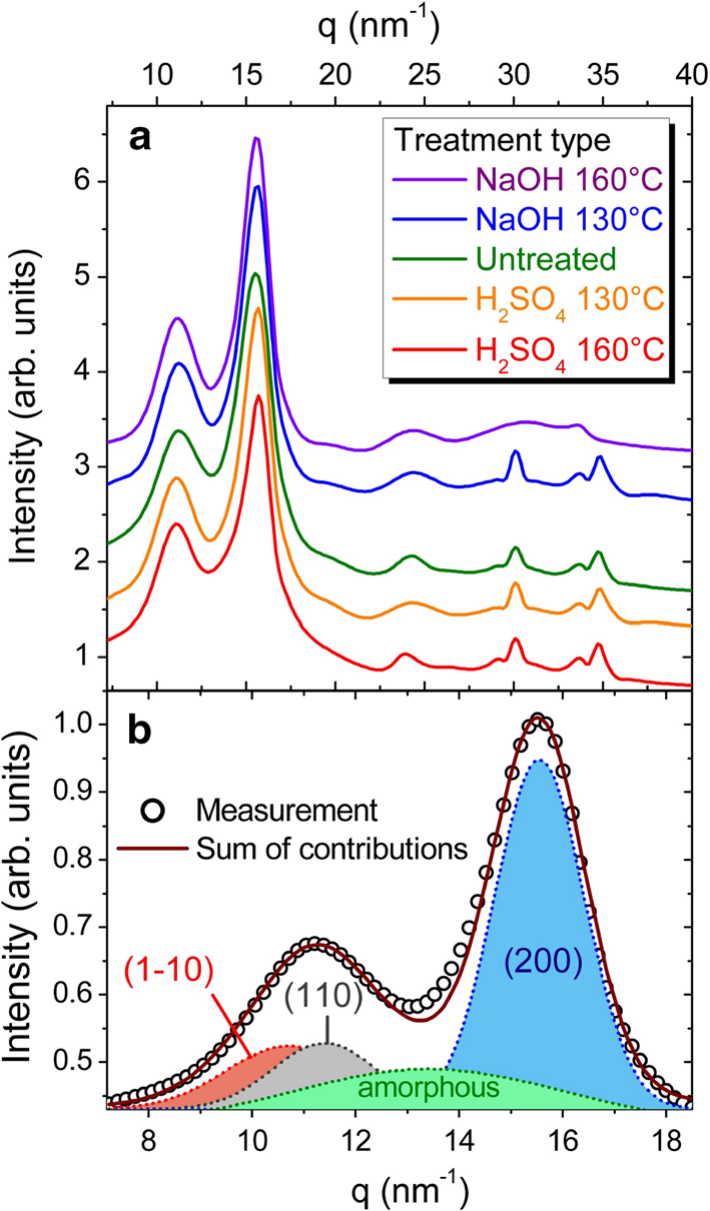
X-ray diffraction of pretreated and *in natura* sugarcane bagasse. Diffractograms for samples in the different treatment conditions are shown in **a**. An example of fits using the components of the most intense peaks observed for *q* < 20 nm^−1^ is shown in **b**. The information extracted from these peaks for all sample conditions is shown in Fig. 6

Modifications in the microstructure of sugarcane bagasse cell walls, observed by AFM, are intrinsically related to the structural conditions of the aggregation of multiple cellulose chains (or, microfibrils) and their close vicinity. To infer their conformation after the discussed pretreatments, X-ray diffraction was carried using both laboratory equipment (Panalytical Empyrean, equipped with a Cu sealed tube producing 8 keV radiation) and synchrotron radiation (XRD1 beamline of the Brazilian Synchrotron Light Laboratory, operating at 12 keV). Despite the larger resolution of the synchrotron source, experiments performed in the abovementioned conditions led to similar results, as depicted in Fig. 5a. Here, the scattering angle is converted into a momentum transfer vector $q\, = \,\left( { 2p/\lambda } \right)\;{ \sin }\left( { 2\theta / 2} \right)$. We could observe in all measurements, including untreated samples and those subjected to NaOH and H_2_SO_4_ treatments, that large peaks are retrieved along the measured reciprocal space region, denoting the presence of structural order within domain sizes of a few nanometers.

Peaks located at large q values (*q* > 20 nm^−1^) can be qualitatively interpreted as indicators of long-range order of cell wall components, these were similar for most of the studied samples, but the NaOH treatment at 160 °C was less organized. At lower *q* values (*q* < 20 nm^−1^), the most intense peaks are observed. These features are related to the $\left( {\overline{110} } \right)$ (110), amorphous and (200) interplanar spacing of cellulose molecules, as discussed in Ref. [45], and can be found centered at q values of approximately 10.8, 11.4, 13.3 and 15.6 nm^−1^. In this interval, fits to the measured intensities can be carried out, as depicted in Fig. 5b, allowing for the extraction of interplanar spacings (given by 2*π*/*q*_c_, where *q*_c_ is the center of a given peak) and average crystallite domain size (given by 2*π*/Δ*q*, where Δ*q* is the peak width).

**Fig. 6:**
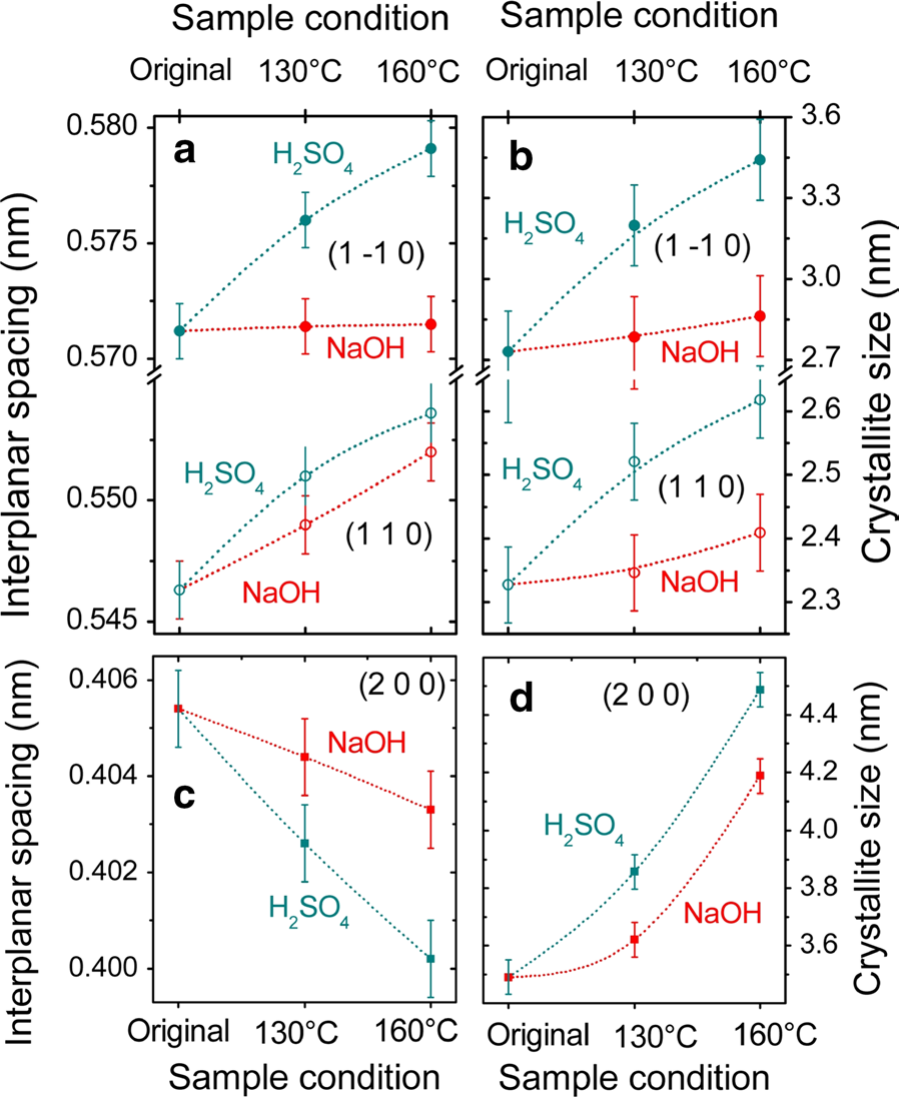
Interplanar spacing and crystallite domain size as a function of the treatment condition. The lattice spacing was evaluated for all sample conditions at the (1− 1 0) and (1 1 0) reflection [panel (**a**)], and at the (2 0 0) reflection [panel (**c**)]. Average crystallite domain sizes extracted from the peak widths are shown in **b** for the (1− 1 0) and (1 1 0) reflections, and panel **d** for the (2 0 0) reflection

Figure 6 summarizes the results for evaluation of interplanar spacing and crystallite domain size carried out at the diffractograms of Fig. 5. We can observe that the interplanar spacing increases in both treatments for the (1 − 1 0) and (1 1 0) planes (Fig. 6a), while a contraction was observed in the (2 0 0) plane (Fig. 6c). In these three reflections, larger changes are seen for the treatment with H_2_SO_4_, for which expansions of 1.4% are observed in Fig. 6a and a contraction of 1.2% is shown in Fig. 6c. We could ascribe the expansion of the interplanar spacing for the (1 − 1 0) and (1 1 0) planes and the contraction for the (2 0 0) plane of the cellulose crystals to the Poisson effect in the cellulose microfibril.

A similar scenario is found when crystallite domain sizes are considered. A more pronounced reciprocal space peak narrowing was observed for the H_2_SO_4_ pretreatment, in comparison with the NaOH pretreatment. This observation is directly related to crystallite domain sizes, found to become larger in the (1 − 1 0) and (1 1 0) planes (Fig. 6b), as well as in the (2 0 0) plane (Fig. 6d). Here, the effects of the NaOH treatment are mild compared to its acid counterpart.

## Discussion

### Sugarcane bagasse cell wall deconstruction by pretreatments

During microwave-assisted acid pretreatment, hemicellulose was hydrolyzed resulting in a mass loss of recovered biomass with a consequent reduction of the cell wall thickness (Fig. 2b). Moreover, the degradation of polymer linkages caused selective lignin removal and relocation in specific ultrastructural layers of the sclerenchyma secondary walls. These changes were followed by a partial or even total detachment of secondary wall layers (Fig. 2b and e). From the AFM results (Fig. 3), a conspicuous enlargement of the width of the cellulose microfibrils (approximately 100 nm) was noticed. Such enlargement might have led to greater contact between cellulose chains which was observed in the XRD results as an expansion of the average crystallite domain size in all directions (Figs. 6b and d). The main indication of the increase in crystallite size, of the order of 30%, is a supra-molecular effect that is evidenced by its comparison with the observed changes in interplanar spacing (that are modified by approximately 1.3%), supporting the physical/mechanical scenario described here.

Alkali can facilitate dissociation of entire wall polymers by breaking hydrogen and covalent bonds, and lignin can be removed [46]. Here, the delignification process of the cell walls broke or weakened the association of cellulose microfibrils, which in turn led to considerable swelling of the secondary cell walls (Fig. 2c). From the FTIR results (Fig. 1), we could confirm that the alkali pretreatment could comprehensively remove lignin from the cell walls. As a result, we could even visualize the micro-fibrillated cellulose structure deep within cell walls as depicted in Fig. 2f. However, a smaller impact on the width of cellulose microfibrils was noticed in the AFM images (Fig. 3e and f). The size of the microfibrils was practically half of that measured for the acid-pretreated fibers. In addition, the XRD results indicated that the delignification process had little effect on the local nanometer-sized arrangement of cellulose molecules (Fig. 6). Both lattice parameters and crystallite domain sizes underwent small changes, except for the increase of (200) crystallite domain size as shown in Fig. 6d.

### Macromolecular ultrastructure of cell walls

Exploitation of lignocellulosic biomass is hampered by our lack of knowledge of the molecular basis for its properties, such as strength and digestibility [47]. It is well-known that fibers consist mainly of cellulose microfibrils in a matrix of intertwined hemicellulose and lignin, called the lignin–carbohydrate complex (LCC) [48–50]. The nature of this interaction remains unclear despite its importance. Here, as far as we know, we provide the first direct evidence of the role of hemicellulose and lignin in the cellulose microfibril assembly.

Our AFM results showed that the average width of the cellulose microfibrils was considerably enlarged with the removal of hemicellulose content (acid pretreatment) when compared to the average value of the delignified sample (alkali pretreatment) (Fig. 3). Moreover, we observed a very organized assembly of cellulose microfibrils in the mw-acid-pretreated SB, whereas less orderly packed microfibrils resulted from the delignification process (Fig. 3). The removal of lignin broke or weakened the association of microfibrils, which in turn led to swelling of secondary cell walls as revealed by TEM images (Fig. 2c).

We hypothesize that hemicellulose is responsible for the rigidity of the lignin–carbohydrate complex that surrounds cellulose microfibrils, holding each microfibril in place. In other words, hemicellulose, instead of lignin, might be the main cellular component responsible for resisting the widening of the spacing between microfibrils, which is considered a key factor for controlling the growth rate of plant cell walls [47]. The cellulose microfibril swelling likely explains a critical mechanism for the enhanced digestibility of acid-pretreated biomass, as it allows for easier penetration of water molecules and enzymes between the cellulose chains. Lignin, however, seems to exhibit stronger adherence to cellulose microfibrils, providing rigid support to the microfibril assemblies by acting as a “glue”.

These observations corroborate with the recent model of the molecular origin of strength and stiffness in lignocellulosic materials proposed by Youssefian and Rahbar [51]. They used molecular dynamics techniques to elucidate the structure, thermodynamic and mechanical properties, and interactions of the bamboo fiber materials. It was reported that the superiority of hemicellulose’s mechanical properties is due to the large number of hydroxyl groups, which increases the hydrogen bond energy density. Lignin’s strong adherence to cellulose microfibrils essentially comes from the large van der Waals energies between lignin and cellulose [51]. This work contributes to a better understanding of the nature of the interactions between cell wall components, which provides the basis of plant recalcitrance to digestibility and deconstruction.

## Conclusions

In summary, we have systematically studied the role of microwave-assisted H_2_SO_4_ and NaOH pretreatments in the deconstruction of plant cell walls from the nano- to micrometer scale. Remarkable details of the cell wall architecture, not previously seen, provide valuable insights to the physical barriers that constrain enzyme accessibility to cellulose, especially at the nanoscale level. This study focused on the highly recalcitrant sclerenchyma cells of sugarcane bagasse.

We found that acid and alkali pretreatments showed marked changes in the structure, architecture, and integrity of cell walls. The degradation of hemicellulose by acid pretreatment promoted removal and relocation of lignin within the secondary cell walls with eventual delamination of the wall layers. Through atomic force microscopy analysis of the macromolecular ultrastructure of cell walls, we observed that the cellulose microfibril was almost double the width of the alkali pretreated SB at the temperature of 160 °C. Such outright enlargement of cellulose microfibrils provides direct evidence that the hemicellulose constituent might be the main barrier for the swelling of cellulose microfibrils.

Through the delignification of sugarcane bagasse cell walls (NaOH pretreatment), we observed that the cellulose microfibril assemblies lost part of their alignment and rigidity along the longitudinal direction. Furthermore, the lignin loss led to a swollen state of the fiber cell walls. These findings provide support to the notion that lignin is important for the rigidity of cell walls. The conclusions from this study should assist in designing more efficient and robust pretreatment processes.

## Methods

### Biomass source and pretreatments

Crushed SB stalks were provided by a sugar and ethanol refinery in Minas Gerais, Brazil. The raw material was previously washed with deionized water and dried in an oven at a temperature of 100 °C for 24 h to remove the moisture. Thereafter, stems were hand-sectioned using a razor blade into short fragments (~ 10 mm) prior to the pretreatment.

A microwave digestion system (Start D, Milestone) with closed reactors was used for sample processing. A total weight of 2.5 g of SB was immersed in 50 mL of 1% m/v H_2_SO_4_ or NaOH solution. The pretreatment was carried out at 800 W (maximum power) at two controlled temperatures (130 and 160 °C), for different reaction severities. The pretreatment was maintained for 30 min. The biomass solid residues resulting from the pretreatments were separated from the liquor by centrifugation, rinsed with deionized water, and dried in an oven at 70 °C for 24 h.

### Fourier transformed infrared spectroscopy

The structural constituents and chemical changes of sugarcane bagasse samples were analyzed using Fourier transformed infrared spectrometry (FTIR). The FTIR spectra were obtained using a powder FTIR spectrometer (Model 1760-X, Perkin-Elmer). The spectra were recorded between 4000 and 500 cm^−1^. Specimens for analysis were prepared by mixing 2 mg of dried sample with 200 mg of KBr [52].

### X-ray diffraction

The crystallite size domain and interplanar spacing of the untreated and pretreated SB samples were measured using an X-ray diffractometer (Empyrean, Panalytical-Philips) using CuKα radiation. The operation voltage and current were maintained at 40 kV and 40 mA, respectively. The 2*θ* range was from 10°–40°, with a step size of 0.5° at a time interval of 7 s.

### Imaging the plant cell wall organization

We performed a multimodal and multiscale approach for the imaging of sugarcane bagasse cell walls before and after microwave acid and alkali pretreatments with different severities. Important aspects of the recalcitrance-related changes of plant structures spanning micro- and nanometer length scales were analyzed. To do this, scanning electron microscopy (SEM), transmission electron microscopy (TEM), and atomic force microscopy (AFM) were applied.

#### Transmission electron microscopy

Samples were fixed by immersion in modified Karnovsky solution (2.5% glutaraldehyde and 2% paraformaldehyde buffered in 0.1 M phosphate buffer) under vacuum. After osmicating and block counterstaining with aqueous uranyl, samples were dehydrated in a graded ethanol series (30, 50, 70, 90%, and twice for 100% ethanol) and acetone; followed by infiltration with increasing concentrations of Spurr resin diluted in acetone (15, 30, 50, 70, 85, 95%, and thrice for 100% resin) under vacuum. Resin blocks with the samples were polymerized at 70 °C for 12 h. Transverse ultrathin sections were obtained in an ultramicrotome (Leica EMUC7) equipped with a diamond knife and mounted on copper grids. The grids were stained with lead citrate. Images were taken with a transmission electron microscope Tecnai G2-12—SpiritBiotwin FEI—120 kV at an accelerating voltage of 80 kV at the Microscopy Center of the Universidade Federal de Minas Gerais (Belo Horizonte, Brazil).

#### Scanning electron microscopy

Images were obtained using a scanning electron microscope (JSM 6360LV, JEOL) using a secondary electron detector. Samples were prepared by cryo-fracturing in liquid nitrogen and sputter-coating with gold with a thickness of approximately 10 nm. Imaging was performed at beam accelerating voltages from 5 to 10 kV.

#### Atomic force microscopy

Fragments of sclerenchyma fibers before and after the pretreatments were deposited onto freshly cleaved mica sheets. The excess water was removed using a flow of nitrogen to minimize possible artifacts generated by humidity and the air-drying procedure, and the samples were imaged immediately. All images of fibrils were acquired by an atomic force microscope (model Cypher ES, Asylum Research) equipped with an AC160TS silicon cantilever (Olympus) operating in the dynamic mode under ambient conditions. Images were zero-order flattened before fibril height determination using the standard algorithm of the AFM system software.
